# Supplementary material for: Nanocapsules of ZnO Nanorods and Geraniol as a Novel Mean for the Effective Control of Botrytis cinerea in Tomato and Cucumber Plants
Source: Plants (Basel). 2023 Feb 28;12(5):1074. doi: 10.3390/plants12051074 (PMC10005723; doi:10.3390/plants12051074)
Supplement: Supplementary file 1 [file plants-12-01074-s001.zip › plants-2228360-supplementary.pdf]

plants

Article

Nanocapsules of ZnO Nanorods and Geraniol as a Novel Means for the Effective Control of *Botrytis cinerea* in Tomato and Cucumber Plants

Panagiota Tryfon [1], Nathalie N. Kamou [2], Akrivi Pavlou [1], Stefanos Mourdikoudis [3,4], Urania Menkissoglu-Spiroudi [2,*] and Catherine Dendrinou-Samara [1,*]

[1] Laboratory of Inorganic Chemistry, Department of Chemistry, Aristotle University of Thessaloniki, 54124 Thessaloniki, Greece; tryfon.giota@gmail.com (P.T.); akrivipavlou@gmail.com (A.P.)
[2] Pesticide Science Laboratory, School of Agriculture, Faculty of Agriculture Forestry and Natural Environment, Aristotle University of Thessaloniki, 54124 Thessaloniki, Greece; nnkamou@gmail.com
[3] Biophysics Group, Department of Physics and Astronomy, University College London, London WC1E 6BT, UK; s.mourdikoudis@ucl.ac.uk
[4] UCL Healthcare Biomagnetics and Nanomaterials Laboratories, 21 Albemarle Street, London W1S 4BS, UK
* Correspondence: rmenkis@agro.auth.gr (U.M.-S.); samkat@chem.auth.gr (C.D.-S.)

Table S1. Values for d-spacing (Å) and diffraction angles (2θ) of the highest intensity reflections for identified phases are present in the analysis separately.

2 Theta (degrees)	d-spacing group (Å)
31.66	2.82
34.36	2.61
36.17	2.48

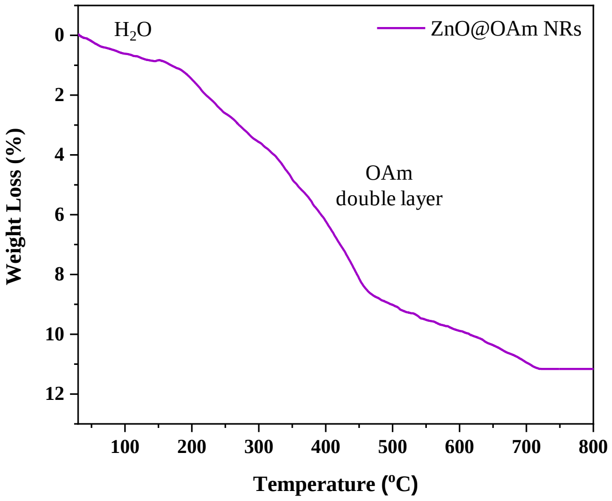

Figure S1. TGA curve for the decomposition of the synthesized ZnO@OAm NRs at the temperature ranges 30–800 °C.

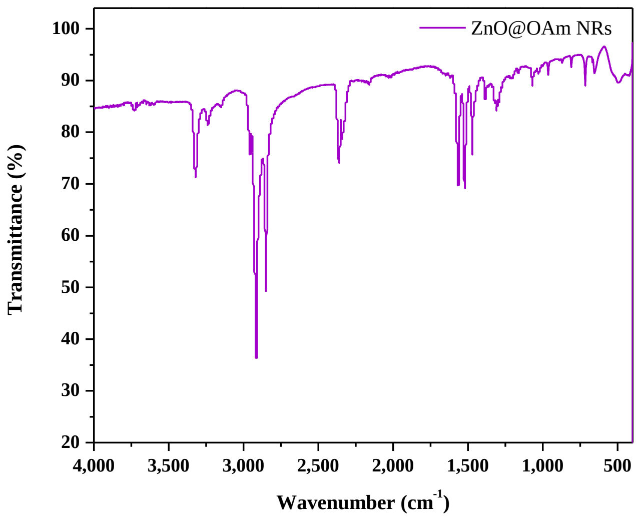

Figure S2. FT–IR spectrum of the ZnO@OAm NRs in the 4,000–450 cm^{-1} region.

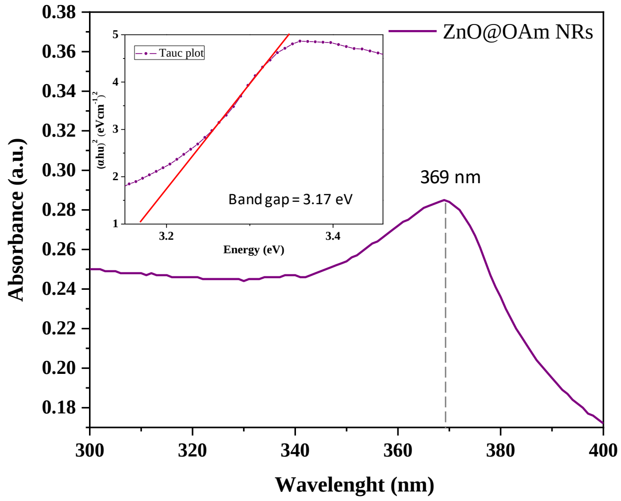

Figure S3. UV–Vis absorption spectrum of the ZnO@OAm NRs with Tauc plots (inset) obtained through standard equations in NPs indicates a band gap of 3.17 eV (B).

Table S2. Pharmacokinetic values (R^2, K_H, N) for ZnOGer1 NCs and ZnOGer2 NCs are derived from zero-order, first-order, Higuchi, and Korsmeyer-Peppas models. (D). The analyses were performed in triplicate. Adjusted R-squared values are also included.

Nanocapsules	Zero-order	First order	Higuchi		Korsmeyer-Peppas	
	R^2	R^2	R^2	K_H	R^2	N
ZnOGer1 NCs 25 °C	0.7388	0.9257	0.9800	9.42	0.9276	0.530
ZnOGer2 NCs 25 °C	0.8589	0.9103	0.9866	13.20	0.8689	0.488
ZnOGer1 NCs 35 °C	0.8776	0.9189	0.9841	11.32	0.8475	0.516

ZnOGer2 NCs 35 °C	0.8957	0.8141	0.9741	14.52	0.9274	0.355

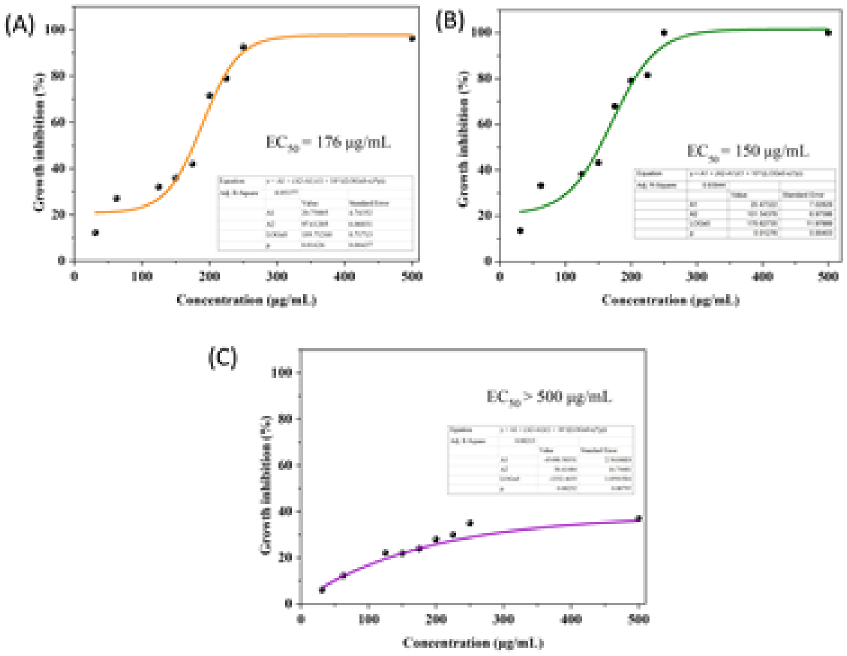

Figure S4. Dose-response growth inhibition curves of B. *cinerea* exposed to nine (9) different concentrations of ZnOGer1 NCs (EC_{50} = 176 μg/mL) (A), ZnOGer2 NCs (EC_{50} = 150 μg/mL) (B), and ZnO NCs (EC_{50} > 500 μg/mL) (C). Each point represents the mean of ten replicates per NCs concentration (two experiments, five replications).
